# Low-Grade Uterine Endometrial Stromal Sarcoma: Prognostic Analysis of Clinico-Pathological Characteristics, Surgical Management, and Adjuvant Treatments. Experience From Two Referral Centers

**DOI:** 10.3389/fonc.2022.883344

**Published:** 2022-06-30

**Authors:** Fulvio Borella, Luca Bertero, Paola Cassoni, Elisa Piovano, Niccolò Gallio, Mario Preti, Stefano Cosma, Domenico Ferraioli, Luca Pace, Luca Mariani, Nicoletta Biglia, Chiara Benedetto

**Affiliations:** ^1^ Division of Gynecology and Obstetrics 1, “City of Health and Science University Hospital”, University of Turin, Turin, Italy; ^2^ Department of Surgical Sciences, University of Turin, Turin, Italy; ^3^ Pathology Unit, Department of Medical Sciences, “City of Health and Science University Hospital”, University of Turin, Turin, Italy; ^4^ Division of Gynecology and Obstetrics 3, “City of Health and Science University Hospital”, Turin, Italy; ^5^ Department of Oncology Surgery, Centre Léon Berard (CLB), Lyon, France; ^6^ Obstetrics and Gynecology University Department, Mauriziano Umberto I Hospital, Turin, Italy

**Keywords:** uterine sarcoma, endometrial stroma sarcoma, morcellation, survival, treatment

## Abstract

**Objective:**

Low-grade uterine endometrial stromal sarcoma (LG-ESS) is a rare tumor characterized by an overall good survival but showing a indolent behavior and a variable risk of recurrence. There is no clear consensus on the optimal management of these tumors and no prognostic or predictive factors have been established. With this study, we evaluated the prognostic relevance of several clinical, surgical, and pathological features in patients affected by LG-ESS to identify risk factors associated with recurrence.

**Methods:**

We retrospectively analyzed 52 LG-ESS cases, treated from January 1st, 1994, to May 31st, 2020, in two referral centers. The relationship between recurrence and clinicopathological characteristics as well as surgical treatment was investigated. Risk of recurrence and disease-free survival (DFS) were estimated by Cox regression and the Kaplan-Meier analysis, respectively.

**Results:**

Of 52 patients with LG-ESS, 8 experienced recurrence (15%). The median follow-up was 100 months (SD ± 96, range: 15–336). By univariate analysis, fragmentation/morcellation, tumor size, FIGO stage, higher mitotic count, presence of necrosis, and lymphovascular space invasion (LSVI) resulted associated with a poorer outcome. Conversely, the surgical modality (laparotomic vs laparoscopic and hysterectomy with bilateral salpingo-oophorectomy vs local excision) and pelvic lymphadenectomy were not. Even the different modalities of adjuvant therapy (hormonal therapy, radiotherapy, and chemotherapy) showed no prognostic significance. Tumor fragmentation/morcellation and higher mitotic count resulted independent prognostic variables at multivariate analysis.

**Conclusions:**

This data supports the avoidance of any type of morcellation if LG-ESS is suspected preoperatively. Higher mitotic count and, possibly, tumor size, advanced FIGO stage, necrosis, and LVSI could be exploited to tailor the adjuvant therapy, but these results need to be confirmed in larger prospective studies.

## Introduction

Uterine sarcomas are rare tumors that originate from either the endometrial connective tissue or the myometrium. These histological entities show a more aggressive behavior and a poorer prognosis compared with the more frequent endometrial cancers of epithelial origin (1). Endometrial stromal sarcomas (ESSs) account for 0.2–1% of all uterine cancers and 6–20% of all uterine sarcomas (2, 3). The latest World Health Organization (WHO) Classification of Tumors of Female Reproductive Organs divides these neoplasms into four distinct groups: Endometrial Stromal Nodule, Low-Grade Endometrial Stromal Sarcoma (LG-ESS), High-Grade Endometrial Stromal Sarcoma (HG-ESS), and Undifferentiated Uterine Sarcoma (UUS) (4). The cornerstone of LG-ESS treatment is represented by total hysterectomy and bilateral salpingo-oophorectomy (BSO) (3, 5) The 5-year and 10-year disease-specific survival rates are 80-90% and 70% respectively, but with an overall risk of recurrence up to 50% (6–9). The role of bilateral pelvic lymphadenectomy, fertility-sparing surgery, and of adjuvant treatments (radiotherapy, hormone therapy, chemotherapy) is still a matter of debate (3, 5). Some authors suggest that intraoperative tumor morcellation increases the risk of recurrence (10, 11), as reported for leiomyosarcomas (12–14), but data addressing this issue are scant. Furthermore, there are no clinical or pathological predictors that can help tailor the allocation of adjuvant therapy. Our study aims to analyze the prognostic value of clinical-pathological characteristics and of the different potential surgical procedures in a cohort of patients affected by LG-ESS treated at two referral centers for gynecological oncology.

## Materials and Methods

This study was conducted retrospectively on 52 patients surgically treated from January 1st, 1994, to May 31st, 2020, in two referral centers (“Città della Salute e della Scienza di Torino - S. Anna Hospital” and “Azienda Ospedaliera Ordine Mauriziano, Torino”), with a histological diagnosis of LG-ESS.

For this study, we considered all naive cases of LG-ESS diagnosed, treated, and histologically confirmed after pathological review with a 12-month follow up available after surgery.

Exclusion criteria were: patients with recurrent LG-ESS at the first visit, with metastatic LG-ESS at the first diagnosis, or without consistent follow-up data. Patients with high-grade (HG)-ESS or other uterine sarcomas (leiomyosarcoma, carcinosarcoma, mixed uterine sarcoma, and undifferentiated sarcoma) were also excluded.

Clinical and pathological data of all patients were extracted from the medical charts and pathology reports including ethnicity, age, number of previous pregnancies, menopausal status, surgical procedure [total hysterectomy with bilateral salpingo-oophorectomy (BSO) vs local excision], surgical approach (laparotomy vs laparoscopy), intraoperative morcellation, bilateral pelvic lymphadenectomy, and pathological FIGO (International Federation of Gynecology and Obstetrics) stage. Morcellation was defined as any manual fragmentation or morcellation (via power morcellator) of the tumor in the abdominal cavity. The tumor stage was determined according to the 2009 FIGO staging for uterine sarcomas (15). All cases were examined by pelvic ultrasound at the time of diagnosis, but for older cases it was not possible to retrieve the initial suspected diagnosis.

Data on patients who underwent adjuvant treatment (hormonal therapy, radiotherapy, or chemotherapy) have been also collected. Since there is no clear consensus on the post-operative management for LG-ESS (3, 16), the adjuvant therapy was decided case by case according to patients’ characteristics (age and comorbidity) and tumor characteristics/stage.

Finally, the following pathological features were considered: tumor maximum size (mm), highest mitotic count per 10 high power field (HPF), presence of coagulative necrosis, and presence of lymphovascular space invasion (LVSI).

All patients underwent periodic follow-up visits every 6 months for 5 years, then yearly. Pelvic examinations and pelvic and abdominal ultrasounds were routinely performed at each visit. When a recurrence was suspected, the diagnosis was confirmed by additional imaging evaluation (abdominal/chest computed tomography, and/or positron emission tomography). Recurrent LG-ESS was defined as the occurrence of new measurable lesion(s) which were then pathologically confirmed.

Survival time was measured from the date of surgery until the last follow-up visit or death for any cause. The specific cause of death was determined by consulting the patients’ medical records or by searching the regional cancer registry. Disease-free survival (DFS) was defined as the time interval between the date of the first diagnosis of LG-ESS and the date of recurrence.

All cases were de-identified, and all clinical-pathological data were accessed pseudonymously.

Written consent was not required considering the retrospective nature of the study. The study was conducted by the Declaration of Helsinki and was approved by our local ethical committee (Protocol number 0119045).

Statistical analyses were performed using IBM^®^ SPSS^®^ v.25 (SPSS Inc., Chicago, IL, USA) software. Data were analyzed descriptively, and categorical variables were represented as counts and percentages, while continuous variables were represented as means with standard deviation (SD) and range. We used the Pearson chi-square test and Student’s t-test to analyze the differences in the distribution of categorical and continuous variables respectively. Survival analysis was done by univariate Cox regression model to calculate the hazard ratios (HRs), while survival curves were estimated by the Kaplan-Meier method and compared with the log-rank test. The continuous variables found to be significant on the t-test were subsequently dichotomized (considering the median as a cut-off) and subsequently analyzed with multivariate Cox regression. All covariates with a p-value < 0.01, based on univariate analysis were included in a multivariate model. Analyses were conducted with a 95% confidence interval (CI), and a p-value of 0.05 was considered statistically significant. All statistical tests were two-tailed.

## Results

We identified 52 patients who underwent surgery for LG-ESS between 1994 and 2020 and met the eligibility criteria.

All patients were Caucasian and the median age at diagnosis was 52 years (SD ± 12, range: 36–92). Most of the patients were multiparous (N=41, 79%) and postmenopausal (N=34, 65%). Regarding surgery, most of the patients were treated with total hysterectomy with BSO (N=48, 92%) by laparotomic approach (N=48, 92%), while in 10 (19%) cases the tumor was morcellated for a presumptive preoperative diagnosis of uterine leiomyoma. Bilateral pelvic lymphadenectomy was performed in 7 patients (13%), but in no case, metastatic lymph nodes were identified.

Most cases were FIGO stage IA (N=25, 48%), followed by IB (N=17, 33%), II (N=7, 13%), and III (N=3, 6%) with a median maximum tumor size of 45 mm (SD ± 41, range 15–200). The median number of mitoses per 10 HPF was 3 (SD ± 4, range 1–19), while coagulative necrosis and LVSI were observed in 6 (11%) and 14 (27%) cases, respectively.

Regarding the adjuvant treatment, 9 patients (17%) received hormonal therapy, 9 (17%) underwent radiotherapy, while only in one case (2%) chemotherapy was administered (4 cycles of doxorubicin and ifosfamid).

The median follow-up duration was 100 months (SD ± 96, range: 15–336) Disease recurrence was observed in 8 cases (15%): 5 patients developed a local recurrence in the pelvic cavity without lymph nodes involvement, two patients had both local (pelvic cavity) and metastatic (lungs) disease recurrence, while only a woman developed lung metastasis without evidence of abdominal disease. The median time of recurrence was 86 months (SD ± 75, range 22-276). Overall, only 3 patients died due to distant secondary metastases from LG-ESS, while 11 patients died from other causes. The remaining 38 patients were alive at the time of the last follow-up.

The 5-year and the 10-year DFS were 94.3% and 84.6%, while the 5-year and the 10-year cancer-specific survival (CSS) were 98% and 94%, respectively.

Between patients with and without disease recurrence, no statistically significant differences were observed in terms of median age, the number of previous pregnancies, menopausal status, surgical procedure and approach, and bilateral pelvic lymphadenectomy. We observed more recurrences in patients who underwent morcellation (60% vs 4.8%, p-value <0.001), with a larger tumor at the first diagnosis (mean largest dimension of 80 mm vs 45 mm, p-value <0.001), and a higher median mitotic count per 10 HPF (3 vs 12, p-value <0.001). According to the histopathological features, the presence of coagulative necrosis and LVSI were associated with recurrence: 50% vs 11% (p-value 0.01) and 50% vs 3% (p-value <0.001), respectively. No significant differences were observed in adjuvant treatments. All data are shown and compared in **Table 1**.

**Table 1:** Clinical and pathological features according to recurrence of 52 patients affected by LG-ESS (in bold significant *p*-values).

	No Recurrence (n = 44)	Recurrence (N = 8)	*p-*value
**Clinical characteristics**			
**Median age (years) SD (range)**	51 ± 12 (31–77)	55 ± 19 (36-84)	0.52
**Number of pregnancies**			
0	8 (73%)	3 (27%)	0.343
≥1	36 (88%)	5 (12%)	
**Menopause**			
No	15 (83%)	3 (17%)	0.85
Yes	29 (85%)	5 (15%)	
**Surgical procedure**			
Hysterectomy + BSO	41 (85%)	7 (15%)	0.499
Myomectomy	3 (75%)	1 (25%)	
**Surgical approach**			
Laparotomy	40 (83%)	8 (17%)	1.00
Laparoscopy	4 (100%)	0 (0%)	
**Morcellation**			
No	40 (95%)	2 (5%)	**<0.001**
Yes	4 (40%)	6 (60%)	
**Lymphadenectomy**			
No	38 (84%)	7 (16%)	0.931
Yes	6 (86%)	1 (14%)	
**Median maximum size (mm)** **(NA = 1)** SD (range)	45 ± 27 (20-120)	80 ± 72 (15-200)	**<0.001**
**FIGO Stage**			
1A	23 (92%)	2 (8%)	0.111
1B	15 (88%)	2 (12%)	
2	2 (67%)	1 (33%)	
3	4 (57%)	3 (43%)	
**Median mitotic count X 10 HPF** SD (range)	3 ± 2.7 (1-9)	12 ± 6 (3-19)	**<0.001**
**Necrosis**			
No	41 (89%)	5 (11%)	**0.01**
Yes	3 (50%)	3 (50%)	
**LVSI**			
No	37 (97%)	1 (3%)	**<0.001**
Yes	7 (50%)	7 (50%)	
**Radiotherapy**			
No	39 (87%)	6 (13%)	0.299
Yes	5 (71%)	2 (29%)	
**Chemotherapy**			
No	44 (86%)	7 (14%)	0.154
Yes	0 (0%)	1 (1%)	
**Hormonal Therapy**			
No	38 (88%)	5 (12%)	0.10
Yes	6 (67%)	3 (33%)	

BSO, bilateral salpingo-oophorectomy; HPF, high-power field; LVSI, lymphovascular space invasion; SD, standard deviation.

By univariate analysis, tumor morcellation (HR 19.46, CI 3.81–99.18, p-value < 0.001), FIGO stage III (HR 7.39, CI 1.21–44.8, p-value 0.03), tumor largest dimension >45 mm (HR 8.85, CI 1.06–73.76 p-value 0.044), mitoses >3 per 10 HPF (HR 4.98, CI 1.004–24.77, p-value 0.049), presence of necrosis (HR 9.57, CI 2.06–44.35, p-value 0.004) and LVSI (HR 28.89, CI 3.54–235.7, p-value 0.002) were found to be associated with DFS (**Table 2**).

**Table 2:** Univariate analysis of the variables associated with LG-EES recurrence (in bold significant *p*-values).

Variable		Univariate analysisHR (95%CI)	*p*-value
**Age**		1.02 (0.97-1.07)	0.46
**Number of pregnancies**	0	1	0.23
	≥1	2.39 (0.57-10.02)	
**Menopause**	No	1	0.81
	Yes	0.84 (0.20-3.52)	
**Surgical procedure**	Hysterectomy	1	0.41
	Myomectomy	0.41 (0.05-3.35)	
**Surgical approach**	Laparotomy	1	0.59
	Laparoscopy	0.04 (0.00-4293)	
**Morcellation**	No	1	**<0.001**
	Yes	19.46 (3.82-99.18)	
**Lymphadenectomy**	No	1	0.72
	Yes	0.67 (0.08-5.72)	
**Size (mm)**		1.02 (1.00-1.03)	**0.006**
**Median dimension (mm)**	≤45	1	**0.044**
	>45	8.86 (1.06.-73.77)	
**FIGO stage**	IA	1	0.675
	IB	1.52 (0.21-10.81)	
	II	9.79 (0.88-108.42)	0.063
	III	7.39 (1.22-44.85)	**0.03**
**Mitotic count X 10 HPF**		1.36 (1.17-1.57)	**<0.001**
**Median mitotic count X 10 HPF (cut-off ≤3) **	≤3	1	**0.049**
	>3	4.99 (1.00-24.77)	
**Necrosis**	No	1	**0.004**
	Yes	9.57 (2.06-44.36)	
**LVSI**	No	1	**0.002**
	Yes	28.89 (3.54-235.75)	
**Radiotherapy**	No		
	Yes	2.02 (0.41-10.04)	0.39
**Chemotherapy**		Not applicable	
**Hormonal therapy**	No	1	0.074
	Yes	3.72 (0.88-15.70)	

BSO, bilateral salpingo-oophorectomy; HPF, high-power field; LVSI, lymphovascular space invasion; HR, hazard ratio.

**Figure 1:**
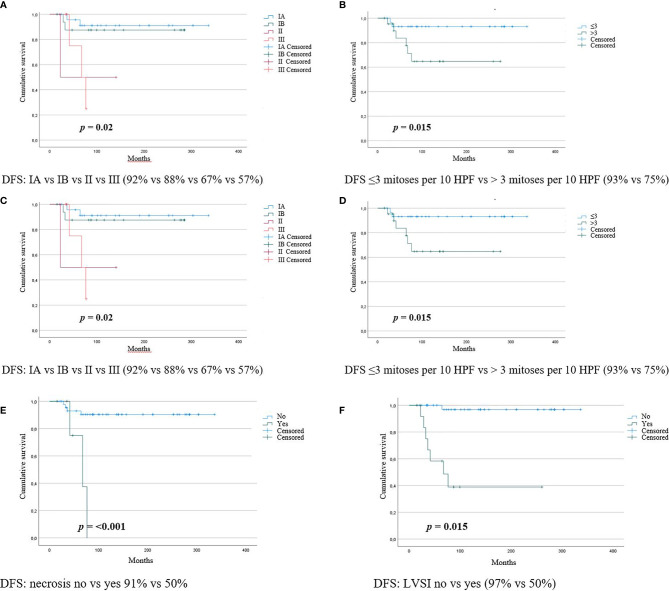
Kaplan-Meier curves for LG-ESS recurrences according to **(A)** morcellation **(B)** tumor size **(C)** FIGO stage **(D)** mitotic count **(E)** necrosis **(F)** LVSI.

The same variables that were significant in the univariate analysis were associated with a reduced DFS after a median follow-up of 100 months when analyzed with the log-rank test (Kaplan-Meier curves are available in **Figure 1**).

Multivariate analysis with Cox-proportional hazard models showed a significantly decreased DFS in case of tumor morcellation (HR 66.1, CI 4.97-881.2, p-value 0.002) and higher mitotic count per 10 HPF (HR 1.41, CI 1.04, 1.91, p-value 0.026).

## Discussion

As mentioned above, the overall cancer specific survival rates of LG-ESS reported in the literature are high but reported recurrence rates are variable and can reach 50% of patients (6–9). Due to the low number of deaths for LG-ESS observed in our series, we decided to focus the analysis on tumor recurrence and ascertain the prognostic significance of multiple clinical, surgical, and pathological features. Furthermore, we evaluated the potential efficacy of adjuvant treatments. Concerning the surgical modality, the first topic of interest was whether total hysterectomy with BSO was associated with lower recurrence rates compared to local excision to preserve fertility. The literature on this topic is very scarce: in a study that included 17 women affected by stage I LG-ESS (stage IA, N = 6; stage IB, N = 11) who underwent conservative surgery, almost all patients (10/11) with stage Ib developed a recurrence (17). In a large cohort of 153 LG-ESS, 19 patients underwent myomectomy with ovary-sparing procedures to preserve fertility, and 4 (21%) developed recurrence (8). Recently, a systematic review focused on fertility-sparing surgery for uterine sarcomas reported a recurrence risk of 54%, however, this result was based on 5 very heterogeneous studies with a relatively low number of patients (18).

In our series, only 4 patients performed a fertility-sparing surgery and in one case a recurrence was observed. No statistically significant difference was detected between this group and patients who underwent hysterectomy with BSO, but this finding should be interpreted with caution due to the limited sample size. Fertility-sparing surgery should be limited to patients with a strong desire for future pregnancies after careful counselling.

The role of systemic lymphadenectomy in LG-ESS treatment is another matter of debate. The percentage of patients in which lymph node metastases were detected after systemic lymphadenectomy ranges from 0% to 9.9% (2, 9, 19), and this procedure does not seem to have an impact on the overall survival (2, 19, 20). More recently, a systematic review including 55 studies on ESS suggested that pelvic and para-aortic lymphadenectomy is not recommended (5). In our cohort, bilateral pelvic lymphadenectomy was performed in 7 patients and no lymph node metastases were observed. Furthermore, amongst the patients who experienced recurrence none occurred in pelvic lymph nodes. Despite the low number of bilateral pelvic lymphadenectomies performed, our data support the omission of this procedure for LG-ESS.

Tumor morcellation is a recognized cause for concern in the treatment of uterine lesions due to the risk of disseminating an unknown malignancy into the pelvic cavity. In particular, in uterine leiomyosarcomas, morcellation is a recognized unfavorable prognostic factor (12–14, 21, 22), but the role of morcellation in LG-ESS is less defined. The first cohort study focusing on the prognostic role of morcellation in LG-ESS analyzed 50 patients with early-stage disease. Of these, 23 underwent tumor morcellation and 27 tumors were not morcellated (10). The authors found that tumor morcellation was significantly associated with poorer DFS (OR 4.03, 5% CI 1.06–15.30; p-value = 0.040) and shorter specific abdominopelvic DFS (OR 5.06, 95% CI 1.02–25.04; p-value = 0.047), but morcellation did not affect the overall survival (10). Conversely, a multicentre study evaluating the prognostic role of morcellation on different types of uterine sarcomas including 14 LG-ESS showed no difference in terms of both DFS and OS for this subtype of tumor (23). The present results are in agreement with the study by Park et al. (10) as even in our series any type of tumor morcellation/fragmentation correlated with a greater risk of recurrence. The Food and Drug Administration (24) and the American College of Obstetricians and Gynecologists (25) stated that laparoscopic power morcellators for the removal of suspected leiomyomas should not be used in postmenopausal women or women older than 50 years, or “candidates for removal of tissue (en bloc) through the vagina or *via* a mini-laparotomy incision.” In-bag morcellation could reduce recurrence rates, but definitive evidence is lacking so far (26). Our data support that any morcellation/fragmentation of LG-ESS (not only power-morcellation) should be avoided if this tumor is suspected.

As expected, the FIGO stage (8, 27–30) and tumor size (29, 31–33) were found to be associated with recurrence also in our study.

Regarding the histopathological features of LG-ESS, we have identified that presence of a mitotic count >3 mitoses per 10 HPF, necrosis, and LVSI are associated with a more aggressive biological behavior. In 1996 Nordal et al (31), who analyzed 48 patients with ESS, suggested a prognostic significance of a higher mitotic count for LG-ESS. Successively, Feng et al. also observed in two different studies (34, 35) that a high mitotic count was associated with an increased risk of recurrence in LG-ESS. Our results reinforce these observations. Similar to higher mitotic count, necrosis has also been found to be associated with recurrence (31, 36). The unfavorable prognostic role of LVSI is well defined in endometrial cancer (37, 38) and a similar association has been reported in LG-ESS (10, 39, 40), but data available to date are limited. In our series, we also found an association between LVSI and DFS. Overall, our results suggest that the classification of LG-ESS should follow strict diagnostic criteria and should be avoided in presence of even focal higher mitotic count. This consideration is in line with the reported possibility of high-grade transformation in LG-ESS as recently reported (41).

LG-ESS frequently expresses receptors for estrogen and progesterone and, on this basis, various hormonal therapy regimens (gestagens, GnRH analogs, or aromatase inhibitors) have been proposed over time, but the results reported so far are based on very heterogeneous small retrospective series and there is no consensus on the effectiveness of these treatments (42).

Adjuvant radiotherapy is another controversial issue. A large retrospective study from the National Cancer Institute’s Surveillance Epidemiology and End Results Program on 1010 patients with ESS diagnosed between 1983 and 2002 found no survival benefit for patients treated with adjuvant radiotherapy (43). In contrast, a reduction of the local failure risk has been reported by a study on uterine sarcomas involving 544 ESSs (44).

Finally, adjuvant chemotherapy seems to offer an advantage in survival for HG-ESS but not for LG-ESS (8, 40); among the patients in the present study, only one received chemotherapy so it is not possible to draw conclusions.

The strengths of this study include the analysis of a relatively large cohort of consecutive patients treated at two referral centers and with extended follow-up data available. Conversely, the major limitations are related to the retrospective design and the potential differences in terms of patients management over the decades, especially considering the lack of standardized guidelines.

## Conclusions

Our results confirm the importance of avoiding any type of morcellation (high-power or manual morcellation) in case of preoperative suspicion of LG-ESS, while the type of surgical procedure does not seem to be related to recurrence risk. We also found that tumor size, advanced FIGO stage, higher mitotic count, necrosis, and LVSI are related to recurrence, thus these characteristics could help tailor the choice of adjuvant therapies, but their further characterization through larger studies is needed.

## Data Availability Statement

The raw data supporting the conclusions of this article will be made available by the authors, without undue reservation.

## Ethics Statement

The studies involving human participants were reviewed and approved by Comitato etico AOU Città della Salute e della Scienza di Torino. Written informed consent for participation was not required for this study in accordance with the national legislation and the institutional requirements.

## Author Contributions

Conceptualization: FB and LB. Literature research: PC, EP, SC, DF, and MP. Data curation: FB, NG, LP, and LM. Statistical analysis: FB.; resources, CB, LM, and MP; writing—original draft preparation: FB and LB. Writing—review, and editing: all authors. Supervision: NB and CB. Resources: NB and CB. All authors have read and agreed to the published version of the manuscript.

## Conflict of Interest

The authors declare that the research was conducted in the absence of any commercial or financial relationships that could be construed as a potential conflict of interest.

## Publisher’s Note

All claims expressed in this article are solely those of the authors and do not necessarily represent those of their affiliated organizations, or those of the publisher, the editors and the reviewers. Any product that may be evaluated in this article, or claim that may be made by its manufacturer, is not guaranteed or endorsed by the publisher.
